# Hypoxia-induced m^6^A demethylation promotes hepatocellular carcinoma progression by preventing the degradation of Gal-1 mRNA

**DOI:** 10.1016/j.gendis.2025.101860

**Published:** 2025-09-18

**Authors:** Xianzhi Gao, Bian Shu, Diguang Wen, Jiao Lu, Hua Song, Ziyi Sheng, Yu You, Zuojin Liu

**Affiliations:** a Department of Hepatobiliary Surgery, The Second Affiliated Hospital of Chongqing Medical University, Chongqing 400010, China; b Department of Hematology, The Second Affiliated Hospital of Chongqing Medical University, Chongqing 400010, China

**Keywords:** ALKBH5, Galectin-1, Hepatocellular carcinoma, HIF-1α, YTHDF2

## Abstract

Increasing evidence indicates that the galectin family played a significant role in tumor progression and is closely related to the hypoxic microenvironment within tumor tissues. However, the regulatory mechanisms behind this process are unexplored. In this study, we found that Gal-1 expression was significantly up-regulated in hepatocellular carcinoma (HCC) tissues and was closely associated with poor prognosis of patients. Intervention of Gal-1 at the cellular level significantly inhibited the malignant phenotype of hepatoma cells. For the first time, we discovered that Gal-1 was regulated by m^6^A modification, ALKBH5 mediated the demethylation of Gal-1 mRNA, and YTHDF2 recognized Gal-1 mRNA and altered its stability. This regulatory process was altered under hypoxic conditions, and hypoxia-inducible factors (HIFs) mediated the regulation of m^6^A modification in hepatoma cells by hypoxia. HIF-1α bound to the promoter region of ALKBH5 and up-regulated ALKBH5 expression, while HIF-2α bound to the promoter region of YTHDF2 and generated negative regulation. *In vivo*, after intervention of Gal-1, reduction of proliferative markers and inhibition of epithelial–mesenchymal transition occurred in the subcutaneous tumor. The use of LNP-siGal-1 also inhibited epithelial–mesenchymal transition.

## Introduction

Primary liver cancer is a highly prevalent and deadly malignancy, with hepatocellular carcinoma (HCC) being the most common subtype, accounting for 75%–85% of cases.^1^ The hypoxic microenvironment is a characteristic feature of most solid tumors, including HCC. This condition arises due to the rapid and uncontrolled proliferation of tumor cells, which leads to increased oxygen consumption. On the other hand, unregulated vascular growth exacerbates the imbalance between oxygen demand and supply.^2^ Hypoxia induces a range of genetic mutations, transcriptional modifications, and subsequent proteomic changes, making hepatoma cells more aggressive.^3^ For instance, hypoxia influences cancer cells, promoting epithelial–mesenchymal transition,^4^ and it also drives senescence,^5^ stem cell-like phenotypes,^6^ and resistance to chemotherapy.^7^

Galectins are a family of endogenous glycan-binding proteins (lectins), characterized by their strong affinity for β-galactoside-containing N-glycans.^8^ Research has shown that galectins often regulate various biological processes in tumors, including cell proliferation,^9^ metastasis,^10^ immune evasion,^11^ resistance to cell death,^12^ and angiogenesis induction.^13^ Notably, galectin-1 (Gal-1) is up-regulated in most tumor tissues and stroma, and it has been identified as a biomarker for poor prognosis in breast cancer,^14^ lung cancer,^15^ prostate cancer,^16^ and melanoma.^17^ These studies suggest that Gal-1 plays a critical role in tumor invasiveness and progression. Gal-1 can crosslink cell-surface glycoconjugates, which trigger a cascade of transmembrane signaling events. Through this mechanism, Gal-1 modulates processes including mitosis, apoptosis, and cell-cycle progression, which enables it to promote the proliferation of tumor cells, including hepatoma. Additionally, through interactions with glycosylated tumor-associated receptors (epidermal growth factor, EGF), transforming growth factor-β (TGFβ) receptors, and cell surface integrins, Gal-1 can trigger epithelial–mesenchymal transition in HCC via non-canonical activation of the Hedgehog pathway, down-regulation of E-cadherins, and induction of αvβ3 integrin-dependent protein kinase B (AKT) signaling. Gal-1 has also been reported to associate with a hypoxic microenvironment. For instance, Diego et al proved that in a hypoxic microenvironment, Gal-1 induced abnormal angiogenesis by interacting with vascular endothelial growth factor receptor 2 (VEGFR2) and transmembrane glycoprotein neuropilin-1 (NRP1), which makes it a pivotal role in resistance to targeted therapy of VEGFR2.^18^ However, despite these findings, the specific association and regulatory mechanism between Gal-1 and hypoxia remain unclear.

m^6^A is the most prevalent post-transcriptional modification in human mRNA and plays a significant role in the progression of HCC.^19^ m^6^A modification is dynamically regulated by methyltransferases, such as methyltransferase 3 (METTL3), METTL14, and Wilms tumor suppressor-1-associated protein (WTAP), as well as demethylases like fat mass and obesity-associated (FTO) and alkane monooxygenase (AlkB) homolog 5 (ALKBH5). These modifications are recognized by specific RNA-binding proteins, including the YTH domain-containing family (YTHDF) proteins and insulin-like growth factor 2 (IGF2) mRNA binding proteins (IGF2BPs). Previous studies have shown that the hypoxic microenvironment influences HCC development through m^6^A modifications in various ways. For example, the increase of ALKBH5 mediated by hypoxia microenvironment in HCC promotes the proliferation and metastasis of HCC cells and the recruitment of programmed cell death ligand 1 (PD-L1)-positive macrophages,^20^ while YTHDF2 inhibits serpin family E member 2 (SERPINE2), leading to the disruption of vascular normalization.^21^ However, the connection between Gal-1 and m^6^A modifications remains unexplored.

In this study, we found that the expression of multiple m^6^A-modifying enzymes in HCC cells was altered under hypoxia, and these changes regulated the post-transcriptional modification process of multiple cancer-related genes, including Gal-1. For the first time, we demonstrated that Gal-1 expression was mediated by m^6^A-dependent control. In this case, ALKBH5 bound to the 5′-UTR region of the Gal-1 mRNA and demethylated a specific site, while the decline of YTHDF2 further enhanced the stability of the Gal-1 mRNA. Furthermore, these regulations were altered by hypoxia-inducible factors (HIFs), the primary sensor and effector of hypoxia. In addition, by encapsulating small interfering RNAs (siRNAs) targeting Gal-1 in lipid nanoparticles (LNPs), we successfully inhibited the malignant progression of HCC in a mouse model of *in situ* liver cancer, suggesting that Gal-1 may be a useful target for HCC immunotherapy. We believe that the revelation of the hypoxia-m^6^A-Gal-1 axis may provide new ideas for the intervention of HCC.

## Methods

### Online data download and processing

Clinical data were obtained from The Cancer Genome Atlas (TCGA), International Cancer Genome Consortium (ICGC), and Hepatocellular Carcinoma Database (HCCDB) portals. Patients were divided into high and low Gal-1 expression groups based on the median expression level. The “limma” package was used to convert the data to transcripts per million (TPM) values for differential analysis, which was used in subsequent analyses.

### Patients

Tumor and adjacent liver tissue samples were collected from 20 patients diagnosed with HCC at the Second Affiliated Hospital of Chongqing Medical University. Informed consent was obtained from all patients, and the study was approved by the Ethics Committee of Chongqing Medical University.

### Cell culture and transfection

The human HCC cell line MHCC-97H was obtained from Zhongshan Hospital (Shanghai, China), while Hep-3B (cat. CL-0102) was purchased from Wuhan Pricella Biotechnology Co., Ltd., Wuhan, China. MHCC-97H cells were cultured in Dulbecco's Modified Eagle Medium containing 10% fetal bovine serum, 100 U/mL penicillin, and 100 μg/mL streptomycin (1% pen-strep). Hep-3B cells were cultured in a specialized medium (Procell, Wuhan, China). Cells subjected to hypoxia were placed in an incubator with 94% N_2_, 5% CO_2_, and 1% O_2_ for 24 h. For cell transduction, when cells reached 70%–80% confluence, 5 μL of siRNA and 4 μL of Lipo8000 (Beyotime Biotechnology, Shanghai, China) were added to 125 μL of basal medium, incubated at room temperature for 20 min, and added to six-well plates. Fresh complete medium was replaced after 8 h, and cells were collected 48 h later for further experiments. Gal-1 lentivirus was purchased from Oligobio (Beijing, China) and added to 24-well plates at 30% confluence. Transduced cells were selected using 2 μg/mL puromycin for 72 h, and fluorescence intensity was observed under a fluorescence microscope to confirm successful transfection. The cells were then cultured in the medium containing 1 μg/mL puromycin.

### CCK-8 assay

HCC cells were inoculated onto 96-well plates overnight. Cell counting kit-8 (CCK-8) reagent (1:10 dilution) was added to each well. Cells were incubated for an additional 2 h and then assayed for 450 nm absorbance using an enzyme-labeled instrument. The experiment was repeated at least three times. The optical density value represents proliferation viability.

### Colony formation test

Single-cell suspensions were inoculated into 6-well plates with approximately 400 cells per well. After 14 days of incubation or after forming clusters of cells visible to the naked eye, cells were fixed in 4% formaldehyde and stained with crystal violet (0.1%). The number of visible colonies was counted. Each experiment was performed three times.

### EdU cell proliferation test

The cells in each group were cultured in six-well plates. When the cells grew to 70% coincidence, 5-ethynyl-2′-deoxyuridine (EdU) at a concentration of 10 M was added, and incubation continued for 4 h. The cells were then fixed in paraformaldehyde. 0.3% Triton X-100 was used for cell permeability. Finally, 4′,6-diamidino-2-phenylindole (DAPI) was added to stain the nucleus. Fluorescent area analysis was performed with the image analysis software FIJI.

### Migration and invasion assays

Cell migration and invasion assays were performed using 8 μm Transwell chambers (NEST, Jiangsu, China). For invasion assays, Matrigel (Solarbio, Beijing, China) was pre-coated on the upper chamber and incubated at 37 °C overnight. Complete Dulbecco's Modified Eagle Medium containing 20% fetal bovine serum was added to the lower chamber, and 5 × 10^4^ cells (97H and Hep-3B) in 200 μL serum-free medium were added to the upper chamber. After 24 h, the chambers were washed with phosphate-buffered saline, fixed with paraformaldehyde, and stained with crystal violet.

### Western blotting and quantitative reverse transcription PCR

For western blotting, cells and tissues were lysed using RIPA buffer (Beyotime Biotechnology, Shanghai, China) and sonicated. After centrifugation at 12,000 *g*, protein concentrations were determined using a BCA kit (Beyotime Biotechnology, Shanghai, China). Gels were prepared using a fast gel preparation kit (Beyotime Biotechnology, Shanghai, China), and electrophoretic transfer to PVDF membranes was performed according to the manufacturer's instructions. Membranes were incubated with primary antibodies at 4 °C overnight, then washed with Tris-buffered saline with Tween 20, and incubated with secondary antibodies at 37 °C for 1 h. Membranes were visualized using high-sensitivity enhanced chemiluminescence (Bio-Rad, USA), and gray values were analyzed using FIJI software. For quantitative reverse transcription PCR, total RNA was extracted using the PrimeScript™ kit (Takara, Japan) according to the manufacturer's instructions, followed by quantitative reverse transcription PCR. All of the Western blotting and PCR assays were repeated at least three times. Antibodies of Gal-1 were purchased from Abcam (ab138513). Antibodies of ALKBH5 (Cat No: 16837-1-AP), HIF-2α (Cat No: 83790-1-RR), and YTHDF2 (Cat No: 24744-1-AP) were purchased from Proteintech. HIF-1α was purchased from Cell Signaling Technology (36169S). The primers used were as follows: Gal-1: AGTCTTCTGACAGCTGGTGC; YTHDF2: GTCAGGGACAAAAGCCTCCG; ALKBH5: GCACGCGCAT; HIF1α: AGAGGTTGAGGGACGGAGAT; Si-Gal-1#1: CCAUCGUGUGCAACAGCAAGG; Si-Gal-1#2: CGGUGACUUCAAGAUCAAAUG; Si-Gal-1#3: GACGGUGACUUCAAGAUCAAA; Si-ALKBH5: GCGCCGUCAUCAACGACUACC; Si-HIF-1α: GCUGGAGUAUGAAGAGCAAGC; Si-HIF-2α: GCUGGAGUAUGAAGAGCAAGC; Oe-Gal-1: LGALS1 PCDH-PURO-3xFlag; Oe-YTHDF2: TK-PCDH-copGFP-T2A-Puro; Oe–HIF–1α: HIF1α PCDH-PURO-3xFlag; Oe–HIF–2α: HIF2α PCDH-PURO-3xFlag.

### Flow cytometry

Digested cells were placed in flow buffer containing 0.05% phosphate-buffered saline. After incubation with antibodies for 30 min, cells were washed three times with phosphate-buffered saline and analyzed for molecular markers using flow cytometry (FlowJo V10 software).

### LNP-siRNA preparation

DLin-MC3-DMA was purchased from Organix (Woburn, Massachusetts, USA), and DSPC, cholesterol, and DMG-PEG2000 were purchased from Avanti Polar Lipids (Alabaster, Alabama, USA). These lipids were mixed at a molar ratio of 50/10/38.5/1.5 in ethanol to form the lipid phase. 2′-O-methyl-modified siRNA was synthesized by Beijing Tsingke Biotechnology Co., Ltd. and dissolved in 25 mM acetate buffer (pH = 4.0) to form the aqueous phase. The two phases were mixed using a Microfluidic chip (Microfluidic ChipShop, Germany) at a volume flow ratio of 3:1 (water/ethanol). The mixture was dialyzed overnight in phosphate-buffered saline to remove ethanol, and the pH was adjusted to 7.4.

### Subcutaneous tumor model and *in situ* carcinoma model in nude mice

MHCC-97H cells transduced with Gal-1 lentivirus (5 × 10^6^ cells/100 μL) were subcutaneously injected into nude mice. These mice were euthanized when tumors reached an appropriate size, and tumor samples were collected. For the orthotopic HCC model, six-week-old male nude mice were fed for one week, and then MCCH-97H cells (1 × 10^6^ cells per mouse) were injected into the left liver lobe. One week later, LNP-siRNA (1 mg/kg) was administered via tail vein injection every three days for 12 days. Tumor size was monitored using *in vivo* imaging, and mice were euthanized for tumor collection. All animal experiments were approved by the Animal Experimentation Ethics Committee of Chongqing Medical University.

### mRNA lifetime experiment

MHCC-97H cells were seeded in six-well plates and treated with 2 μg of actinomycin D. Total RNA was extracted at 0, 2, 4, and 8 h using TRIZOL, and Gal-1 mRNA abundance was measured by quantitative reverse transcription PCR.

### RNA fluorescence *in situ* hybridization

Fluorescence *in situ* hybridization was performed using an mRNA *in situ* hybridization kit (Shanghai GenePharma Co., Ltd., China). Probe buffer was added to the cell samples, and they were then co-incubated overnight. Gal-1 mRNA co-localization with ALKBH5 or YTHDF2 was observed under a confocal microscope, and FIJI software was used for quantitative analysis.

### Immunohistochemistry and immunofluorescence staining

Human liver cancer tissues or mouse subcutaneous tumor tissues were embedded in paraffin and sectioned. After antigen retrieval, the sections were blocked and incubated overnight with primary antibodies. For immunohistochemistry, the sections were washed with phosphate-buffered saline and incubated with secondary antibodies at room temperature for 1 h, followed by DAB and hematoxylin staining. Images were captured, and staining intensity was evaluated using the IRS immunohistochemistry scoring system. For immunofluorescence, after phosphate-buffered saline washing, sections were incubated with fluorescent secondary antibodies at room temperature for 1 h, followed by DAPI staining and imaging.

### RNA immunoprecipitation

MHCC-97H and Hep-3B cells (2 × 10^7^) were collected and processed using the Magna RIP™ RNA-binding protein immunoprecipitation kit (Beyotime Biotechnology, Shanghai, China) according to the manufacturer's instructions. Cells were lysed, and lysates were divided into input, IgG, and immunoprecipitation groups. Antibodies or normal rabbit IgG were added and subjected to incubation at 4 °C overnight, followed by incubation with magnetic beads for 3–5 h. RNA-protein complexes were eluted, RNA was extracted using TRIZOL, and quantitative reverse transcription PCR was performed.

### CUT&RUN assay

MHCC-97H cells (1 × 10^5^) were collected, and fragmented DNA was processed using the Hyperactive pG-MNase CUT&RUN Assay Kit (Vazyme, Nanjing, China). DNA was collected, and quantitative reverse transcription PCR was performed using primers designed for the gene promoter region.

### Dual luciferase assay

MHCC-97H cells were seeded in six-well plates. When cells reached 70%–80% confluence, NC or siRNA was transfected. Two days later, luciferase reporter plasmids (wt or mut) corresponding to the target gene sequence (1 μg/mL) were transfected. After 48 h of incubation, luciferase activity was measured using the Dual Luciferase Reporter Gene Assay Kit (Beyotime Biotechnology, Shanghai, China) and compared between the siRNA-transfected and NC groups.

### Statistical analysis

Quantitative data were analyzed using one-way analysis of variance (ANOVA) or Student's *t*-test. Categorical variables were compared using the chi-square or Fisher's exact test. Survival analysis was performed using the Kaplan–Meier method. Statistical significance was set at *p* < 0.05 (∗*p* < 0.05, ∗∗*p* < 0.01, and ∗∗∗*p* < 0.001; ns, not significant). All data were analyzed using SPSS 24.0 and PRISM 9.0 software.

## Results

### High Gal-1 expression is associated with HCC progression

Combined analysis of multiple databases, including HCCDB, ICGC, and TCGA, revealed that patients with high expression of Gal-1 had poorer overall survival (Fig. 1A–C). Analysis of differentially expressed Gal-1 in HCC based on ICGC and TCGA showed significant differences (Fig. 1D and E). Immunohistochemical analysis (Fig. 1F; Fig. S1A) and Western blotting (Fig. 1G; Fig. S1B) results confirmed that Gal-1 expression was significantly elevated in tumor tissues compared with adjacent normal tissues in HCC patients. These findings suggest that the high expression of Gal-1 contributes to the malignant progression of HCC.

**Figure 1:**
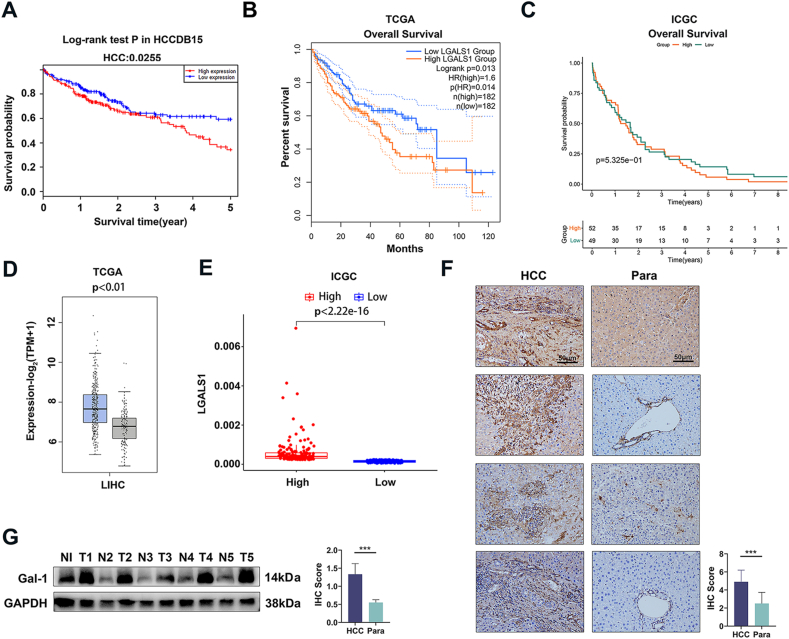
High Gal-1 expression is associated with hepatocellular carcinoma (HCC) progression. **(A)** Kaplan–Meier survival analysis was performed using the HCCDB database, showing that high Gal-1 expression correlates with shorter overall survival compared with the low-expression group. **(B)** The overall survival (OS) of patients with high (*n* = high) and low (*n* = low) Gal-1 expression was compared using Kaplan–Meier survival analysis based on data from the TCGA database. **(C)** The OS of patients with high (*n* = high) and low (*n* = low) Gal-1 expression was compared using Kaplan–Meier survival analysis based on data from the ICGC database. **(D)** The expression of Gal-1 was analyzed in HCC tissues (*n* = 369) and normal tissues (*n* = 160) using data from the TCGA database. **(E)** The analysis revealed significant differences in Gal-1 expression between the two groups. Comparing Gal-1 expression in HCC and adjacent tissues using the ICGC database further confirmed the differential expression observed in the TCGA dataset. **(F)** Immunohistochemical staining for Gal-1 was performed on 20 pairs of HCC tissues and adjacent normal tissues. The staining results demonstrate a significant increase in Gal-1 expression in tumor tissues compared with adjacent normal tissues. **(G)** Western blotting analysis was conducted to compare Gal-1 expression in 10 pairs of HCC tissues and adjacent normal tissues. The results show a marked increase in Gal-1 protein levels in HCC tissues.

### Gal-1 expression modulates proliferation and epithelial–mesenchymal transition in HCC cells

Subsequently, Gal-1 was knocked down in the hepatoma cell lines MHCC-97H and Hep-3B (Fig. 2A). Following this, CCK8 assays (Fig. 2B), colony formation assays (Fig. 2C), and EdU probe assays (Fig. 2D) were conducted to evaluate cell proliferation. The results indicated that cell proliferation was significantly inhibited in the Gal-1 knockdown group compared with the control group. Furthermore, the knockdown of Gal-1 in HCC cell lines MHCC-97H and Hep-3B resulted in decreased invasiveness and migration capabilities (Fig. 2E). Western blotting analysis revealed an increase in E-cadherin expression, while N-cadherin and Vimentin expression were found to be reduced (Fig. 2F). Furthermore, overexpression of Gal-1 (Fig. 3A) enhanced the proliferative (Fig. 3B–D) and metastatic (Fig. 3E) capacities of HCC cells, with concomitant reverse alterations in the protein expression of epithelial-mesenchymal transition-associated markers (N-cadherin, E-cadherin, and Vimentin) compared with Gal-1 knockdown (Fig. 3F).

**Figure 2:**
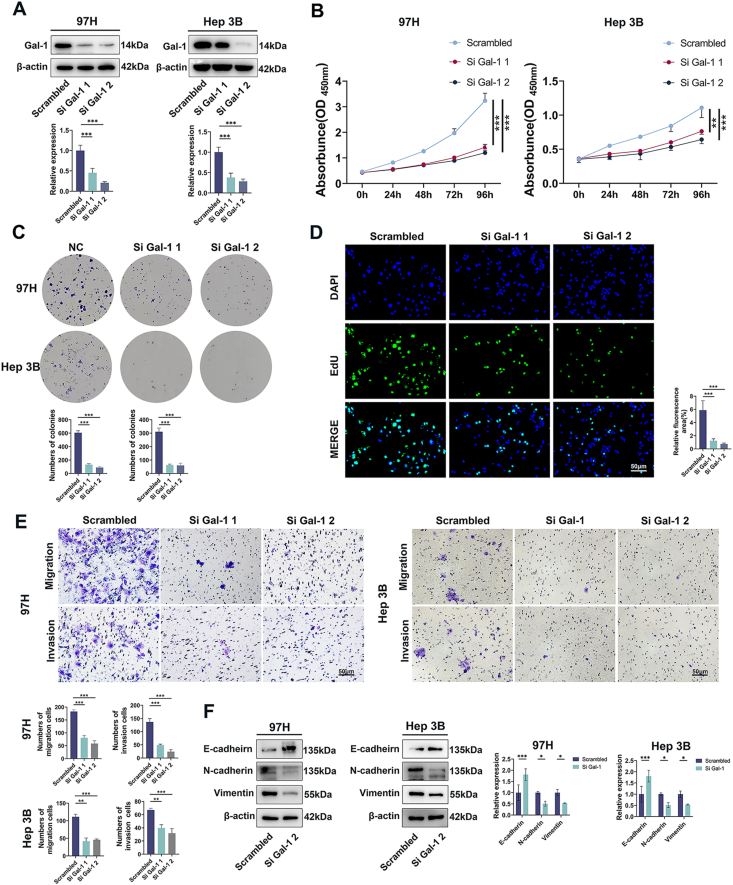
Gal-1 knockdown inhibited the proliferation and epithelial–mesenchymal transition of HCC cells. **(A)** Gal-1 was knocked down by siRNA. **(B)** CCK8 assays were performed on HCC cell lines, and the proliferation process of Gal-1 knockdown cells was significantly slowed down. **(C)** Cell colony formation assay showed fewer colonies Si Gal-1 group (*n* = 3). **(D)** The EdU probe was used for MHCC-97 cells to detect the effect of Gal-1 overexpression on HCC proliferation rate (*n* = 3). **(E)** Migration and invasion assays were performed on HCC cell lines (*n* = 3). Knockdown of Gal-1 significantly reduced the invasive and migratory capabilities of the cells. **(F)** Western blotting analysis revealed that Gal-1 knockdown led to an increase in E-cadherin expression and a decrease in N-cadherin and Vimentin expression.

**Figure 3:**
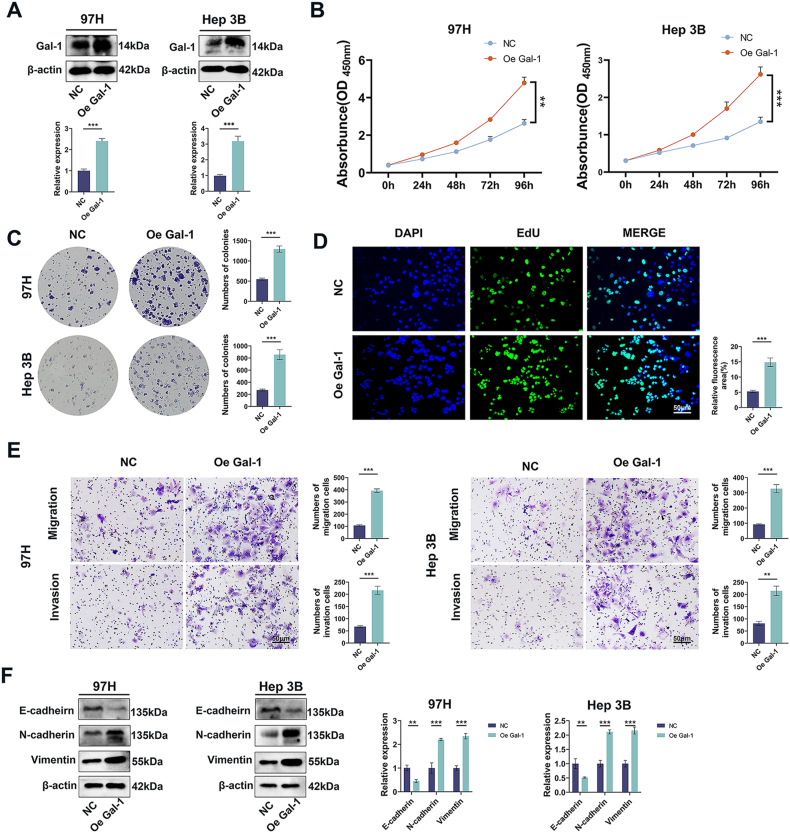
Gal-1 overexpression enhanced the proliferation and epithelial–mesenchymal transition of hepatocellular carcinoma cells. **(A)** Western blotting showed overexpressed Gal-1. **(B)** CCK8 assay demonstrated that the proliferation of HCC cells was significantly up-regulated after overexpressing Gal-1 (*n* = 3). **(C)** Cell colony formation assay showed more colonies in the Oe Gal-1 group (*n* = 3). **(D)** The EdU probe was used to detect the effect of Gal-1 overexpression on HCC proliferation rate (*n* = 3). **(E)** Migration and invasion assays were performed on HCC cell lines (*n* = 3) after overexpressing Gal-1 (*n* = 3). **(F)** Western blotting analysis revealed that overexpressing Gal-1 decreased E-cadherin expression and increased N-cadherin and Vimentin expression.

### High expression of Gal-1 is associated with m^6^A modification

**Figure 4:**
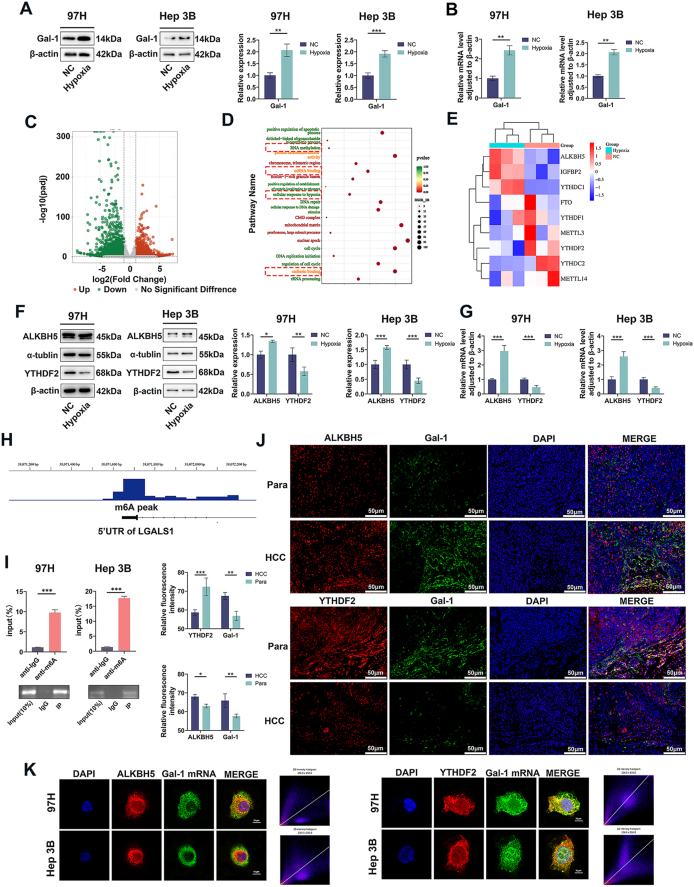
High expression of Gal-1 is associated with m^6^A modification. **(A)** Western blotting analysis was performed to examine the effect of hypoxia on Gal-1 protein expression in HCC cells. **(B)** Quantitative real-time PCR was used to analyze the expression of Gal-1 mRNA in HCC cells under hypoxic conditions. **(C)** Whole transcriptome sequencing of hypoxia-treated MHCC-97H cells identified 4496 differentially expressed genes (DEGs) (*n* = 3). **(D)** The bubble plot displays the pathways altered by hypoxia, highlighting signaling pathways of RNA methylation, mRNA binding, cadherin binding, and cellular response to hypoxia. **(E)** The transcriptome sequencing data of MHCC-97H cells under hypoxia revealed significant changes in the expression of m^6^A-modifying enzymes. **(F)** Western blotting analysis indicated that hypoxia significantly altered the expression of ALKBH5 and YTHDF2. **(G)** Quantitative real-time PCR results showed significant changes in ALKBH5 and YTHDF2. **(H)** Analyzing the Gene Expression Omnibus data series, it was found that there was an m^6^A site near the peak at the Gal-1 5′-UTR region. **(I)** RNA immunoprecipitation and agarose gel electrophoresis assay were conducted to demonstrate the m^6^A modification of Gal-1 (*n* = 3). **(J)** Immunofluorescence analysis showed co-expression of ALKBH5 or YTHDF2 with Gal-1 in HCC and adjacent normal tissues (*n* = 3). **(K)** Fluorescence *in situ* hybridization analysis showed co-localization of ALKBH5 or YTHDF2 with Gal-1 mRNA in HCC Cells (*n* = 3).

Previous studies have shown that the hypoxic microenvironment plays a crucial role in liver cancer progression,^7^ and as a hypoxia-responsive gene, Gal-1 is also affected by hypoxia. We treated HCC cells with hypoxic conditions and found that Gal-1 expression significantly increased (Fig. 4A and B); HCC cells showed a stronger ability of invasion and metastasis (Fig. S1C and S1D). It was reported that the hypoxia marker HIF-1α regulated Gal-1 in retinal pigment epithelium cells,^22^ and correlation analysis from the TCGA database also verified the association between HIF-1α and Gal-1 (Fig. S1E), but our experiments revealed no significant binding of HIF-1α to the Gal-1 promoter region in HCC cells (Fig. S1F). This led us to hypothesize that there may be some other mechanisms. To find the mechanism of hypoxia regulating Gal-1, we performed transcriptome sequencing in hypoxia-treated MHCC-97H cells, and identified 4496 differentially expressed genes (Fig. 4C). Given our group's long-standing research on m^6^A modifications,^23^ our Gene Ontology (GO) analysis revealed alterations in mRNA binding and RNA methylation pathways (Fig. 4D). The expression of a variety of m^6^A-related enzymes changed, including ALKBH5, IGFBP2, YTHDC1, YTHDC2, YTHDF2, and METTL14 (Fig. 4E). Of these, IGFBP2, ALKBH5, and YTHDF2 were highly correlated with Gal-1 (Fig. S2A). By analyzing survival data from TCGA and ICGC databases, we found that ALKBH5 and YTHDF2 were associated with poor prognosis of HCC, while IGFBP2 was not (Fig. S2B). Therefore, ALKBH5 and YTHDF2 were selected for follow-up experiments. Western blotting and quantitative PCR results showed that ALKBH5 expression significantly increased while YTHDF2 expression significantly decreased (Fig. 4F and G). Analysis of the Gene Expression Omnibus data series to elucidate the m^6^A enzyme-Gal-1 binding mechanism revealed putative binding sites within the Gal-1 mRNA 5′-UTR (Fig. 4H). Subsequent RNA immunoprecipitation-quantitative PCR experiments confirmed the presence of m^6^A modifications on Gal-1 mRNA (Fig. 4I). To find their association with Gal-1, we performed co-expression fluorescence staining of Gal-1 and YTHDF2 or ALKBH5 in HCC and adjacent tissues (Fig. 4J). We found that the co-expression of ALKBH5 with Gal-1 was higher in HCC tissues than in adjacent normal tissues, while YTHDF2 showed the opposite phenomenon. Fluorescence *in situ* hybridization experiments in MHCC-97H and Hep-3B cells revealed the same high degree of co-localization between ALKBH5/YTHDF2 and Gal-1 mRNA (Fig. 4K). Taken together, these findings suggest that the expression of Gal-1 may be regulated by ALKBH5 and YTHDF2.

### ALKBH5 and YTHDF2 cooperatively regulate Gal-1 expression

**Figure 5:**
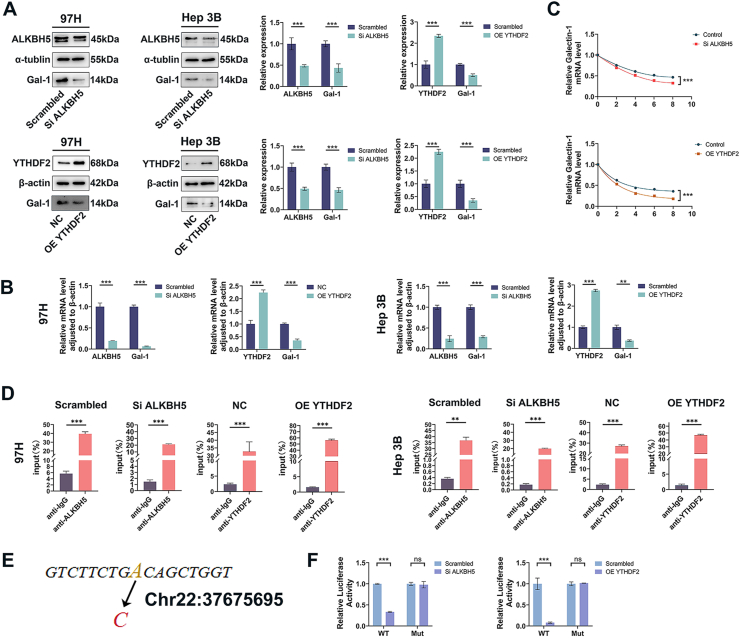
ALKBH5 and YTHDF2 cooperatively regulate Gal-1 expression. **(A)** Western blotting analysis showed reduced Gal-1 protein expression after ALKBH5 knockdown or YTHDF2 overexpression. **(B)** Quantitative real-time PCR analysis demonstrated a significant reduction in Gal-1 mRNA expression following the intervention of ALKBH5 or YTHDF2. **(C)** Quantitative real-time PCR analysis indicated that intervention of ALKBH5 or YTHDF2 reduced the stability of Gal-1 mRNA, leading to a shorter mRNA half-life. **(D)** RNA immunoprecipitation experiments confirmed that ALKBH5 and YTHDF2 bound to Gal-1 mRNA in HCC cells. Upon intervention of ALKBH5 and YTHDF2, the binding level to Gal-1 mRNA was significantly reduced. **(E)** The SRAMP and RMVar online tools were used to predict potential m^6^A modification sites on Gal-1 mRNA. **(E)** Dual-luciferase reporter assay based on the predicted m^6^A sites from (B) demonstrated that ALKBH5 was unable to regulate the mutant Gal-1 5′-UTR.

Next, we found that the knockdown of ALKBH5 or the overexpression of YTHDF2 inhibited the expression of Gal-1 in hepatoma cells, which confirmed our hypothesis (Figure 4, Figure 5B). Since the regulation of m^6^A modification on post-transcriptional modification mainly affected mRNA stability, actinomycin D experiment was carried out to detect the half-life of Gal-1 mRNA. The results showed a decline in mRNA stability, explaining the inhibition of Gal-1 (Fig. 5C). Further RNA immunoprecipitation-quantitative PCR experiments confirmed that ALKBH5 and YTHDF2 bound to Gal-1 mRNA, and the knockdown of ALKBH5 or the overexpression of YTHDF2 changed their binding level with Gal-1 (Fig. 5D). At last, bioinformatic prediction in website SRAMP(http://www.cuilab.cn/sramp) and RMVar (https://www.rmvar.renlab.org/) identified potential binding sites of ALKBH5 and YTHDF2 on Gal-1 mRNA (Fig. 5E; Fig. S2C), and dual-luciferase reporter assays with site-directed mutagenesis validated the specific binding sites (Fig. 5F). These results demonstrate that ALKBH5 and YTHDF2 regulate Gal-1 mRNA stability by modulating m^6^A methylation levels, which subsequently affects Gal-1 protein expression.

### HIFs regulate the expression of ALKBH5 and YTHDF2

**Figure 6:**
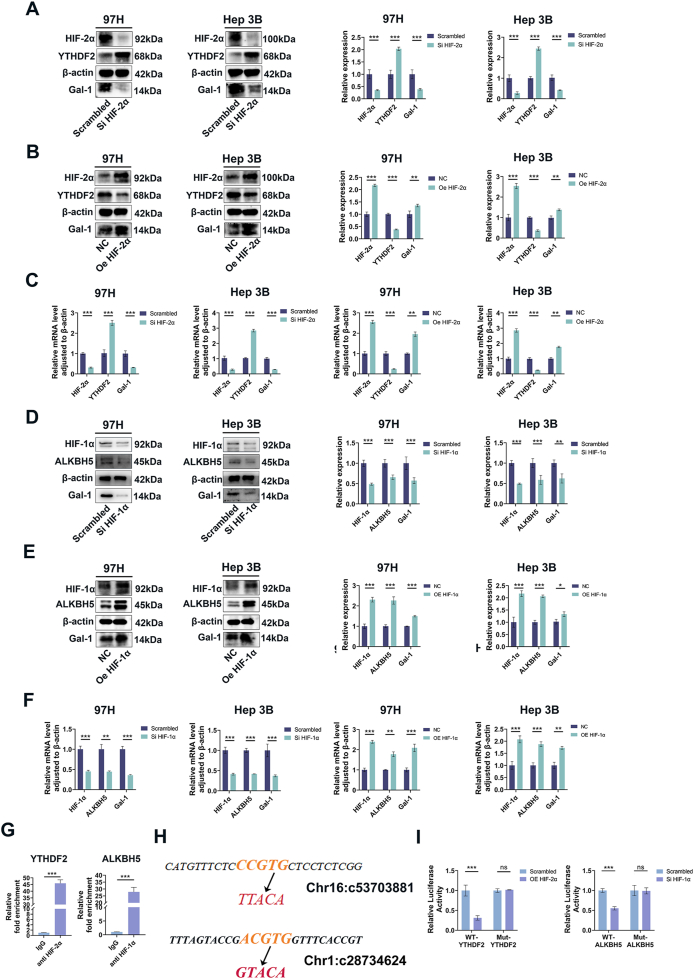
HIFs regulate the expression of ALKBH5 and YTHDF2. **(A)** Western blotting analysis was performed after HIF-2α knockdown using siRNA in HCC cells. The results showed that knockdown of HIF-2α led to a significant rise of YTHDF2, and reduction of Gal-1. **(B)** Western blotting analysis was performed after HIF-2α overexpression in HCC cells. **(C)** Quantitative real-time PCR experiment was performed after HIF-2α knockdown or overexpression. **(D)** Western blotting analysis showed reduction of ALKBH5 and Gal-1 after HIF-1α knockdown. **(E)** Western blotting analysis was performed after HIF-1α overexpression in HCC cells. **(F)** Quantitative real-time PCR experiment was performed after HIF-1α knockdown or overexpression. **(G)** CUT&RUN experiments demonstrated that HIF-1α functioned as a transcription factor by binding to the promoter regions of ALKBH5, and YTHDF2 was regulated by HIF-2α. **(H)** Using the JASPAR database, potential HIF binding sites on the promoters of ALKBH5 or YTHDF2 were predicted. **(F)** Site-directed mutagenesis of the predicted HIF binding sites on the ALKBH5 promoters (from D) was performed, which confirmed that HIF-1α was unable to bind to the mutated ALKBH5, and HIF-2α could not regulate Mut-YTHDF2.

Though we have established the regulatory roles of ALKBH5 and YTHDF2 in Gal-1 expression, it remains unclear how hypoxia changes this process. It has been reported that the reduction of YTHDF2 in hypoxic environments is mediated by HIF-2α. After our intervention on HIF-2α (Fig. 6A–C), we reconfirmed this theory. However, through the correlation analysis of the TCGA database, ALKBH5 has no correlation with HIF-2α, but is significantly correlated with HIF-1α instead (Fig. S2D). To explore this, we knocked down HIF-1α and observed a reduction of ALKBH5 through quantitative PCR and Western blotting, while overexpressing HIF-1α showed the opposite effect (Fig. 6D–F). As a key factor in cellular response to hypoxic stress, HIFs function mainly by regulating the promoter region of downstream proteins as a transcription factor. So, we conducted CUT&RUN experiments to reveal whether this modulation was present in our study. Figure 6G showed that HIF-1α bound to the promoter regions of ALKBH5 when YTHDF2 was regulated by HIF-2α. Using JASPAR (https://jaspar.elixir.no/), we predicted the binding site in the promoter area of ALKBH5 and YTHDF2 (Fig. 6H; Fig. S2E). After mutating these sites, dual-luciferase reporter assays revealed that HIF-1α lost its control of mut-ALKBH5, and HIF-2α could not regulate mut-YTHDF2, either (Fig. 6F). These findings showed that under hypoxic conditions, HIFs increased in hepatoma cells and altered the expression of m^6^A-related enzymes, including ALKBH5 and YTHDF2. HIF-1α mediated the increase of ALKBH5, and HIF-2α reduced the expression of YTHDF2. These changes jointly mediated the demethylation of downstream Gal-1 mRNA, which further promoted the development of liver cancer.

### Targeting Gal-1 expression inhibits subcutaneous tumor growth and orthotopic liver cancer progression in mice

**Figure 7:**
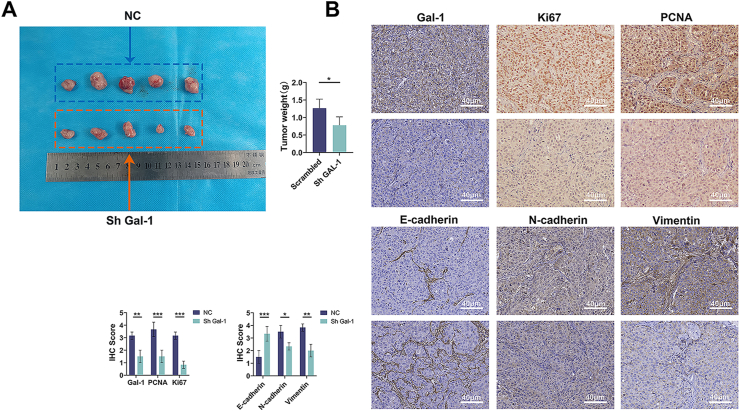
Targeting Gal-1 expression inhibits subcutaneous tumor growth. **(A)***In vivo* experiments demonstrated that knockdown of Gal-1 significantly inhibited the growth of MHCC-97H cells in nude mice. **(B)** Immunohistochemical analysis revealed that Gal-1 knockdown led to an increase in E-cadherin expression and a decrease in Ki67, PCNA, N-cadherin, and Vimentin expression.

**Figure 8:**
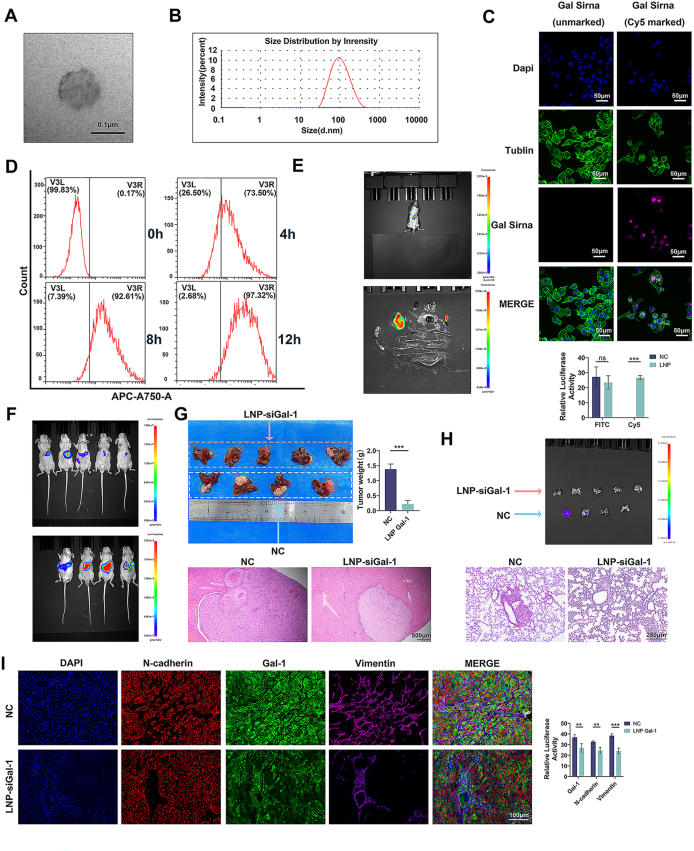
Targeting Gal-1 expression inhibits orthotopic liver cancer progression in mice. **(A)** Transmission electron microscope images were captured to visualize the structure of LNP-siGal-1 after its assembly. **(B)** Particle size analysis of LNP-siGal-1 was performed to assess the size distribution of the assembled nanoparticles. **(C)** Confocal immunofluorescence microscopy confirmed the successful internalization of LNP-siGal-1 into MHCC-97H cells. **(D)** Flow cytometry was used to track the internalization of LNP-siGal-1 into MHCC-97H cells. **(E)** Biodistribution analysis showed the accumulation of LNP-siGal-1 in different organs following systemic administration. **(F)***In vivo* imaging of mice treated for 12 days with LNP-siGal-1 demonstrated significant differences in fluorescence signals, indicating effective delivery and accumulation of the nanoparticles in liver. **(G)** LNP-siGal-1 treatment significantly reduced the tumor volume in a mouse orthotopic liver tumor model. Hematoxylin-eosin staining of HCC tissues from mice treated with LNP-siGal-1 and LNP-NC showed marked differences in tumor architecture. **(H)***In vivo* imaging and hematoxylin-eosin staining of HCC lung tissues indicated that LNP-siGal-1 reduced tumor metastasis. **(I)** Immunofluorescence analysis of HCC tissues confirmed that treatment with LNP-siGal-1 resulted in reduced Gal-1 expression. Additionally, the expression levels of epithelial–mesenchymal transition markers Vimentin and N-cadherin were significantly decreased in the LNP-siGal-1 group.

*In vivo*, firstly, we conducted a subcutaneous tumor formation assay in immunodeficient mice by transducing the HCC cell line MHCC-97H. Knockdown of Gal-1 significantly inhibited tumor growth, with the weight of tumors in the Gal-1 knockdown group being markedly lower than that in the control group (Fig. 7A). Immunohistochemistry indicated the expression of proliferating cell nuclear antigen (PCNA), Ki67, N-cadherin, and Vimentin wwas reduced, while E-cadherin expression was increased after Gal-1 was knocked down (Fig. 7B). Additionally, we developed lipid-siRNA nanoparticles (LNPs) encapsulating siGal-1, validated by transmission electron microscopy and particle size analysis (Fig. 8A and B). Immunostaining and flow cytometry confirmed that LNP-siGal-1 was transduced in MHCC-97H cells (Fig. 8C and D). *In vivo* imaging following tail vein injection showed significant accumulation of LNP-siGal-1 in the mouse liver (Fig. 8E). These results validated the feasibility of LNP-siGal-1 as a targeted therapy. Then, we established an *in situ* liver cancer model in nude mice and randomly divided them into the NC group and LNP group, and injected LNP-siGal-1 or LNP-NC, respectively, within 12 days (Fig. 8F). Figure 8G and H showed that tumor weights were significantly inhibited, and LNP-siGal-1 treatment markedly suppressed the pulmonary metastases. Immunofluorescence analysis indicated that LNP-siGal-1 significantly reduced Gal-1 levels *in vivo*, which, in turn, inhibited epithelial–mesenchymal transition (Fig. 8I). These findings highlight the potential of Gal-1 as a therapeutic target in HCC.

## Discussion

Currently, surgical resection is the preferred treatment for HCC, and it is often effective for early-stage cases.^24^ However, due to the asymptomatic nature of HCC in its early stages, many patients miss the optimal window for surgery. Patients diagnosed at intermediate or advanced stages have a low chance of surgical resection, which leads to poor prognosis.^25^ Therefore, identifying multiple genetic targets to explore more effective treatments is crucial. In this study, we found that Gal-1 showed a significant increase in HCC, which is associated with the hypoxic microenvironment. Based on previous studies^22^^,^^26^^,^^27^ and TCGA data analysis, we believe that HIFs may be involved in the regulation of Gal-1. Our integrated molecular studies establish, for the first time, m^6^A-dependent regulation of Gal-1, mediated by the HIF-1α and HIF-2α-dependent pathway involving ALKBH5 and YTHDF2.

First, through the joint analysis of TCGA, ICGC, HCCDB, and other databases, we found that there were significant differences in the expression of Gal-1 between HCC and adjacent normal tissues, and high expression of Gal-1 was often associated with worse overall survival. These data highlight the critical role of Gal-1 in HCC progression. In terms of mechanism, according to a previous study, Gal-1 overexpression up-regulates αvβ3 integrin, which triggers the hyperactivation of focal adhesion kinase (FAK)/phosphoinositide 3-kinase (PI3K)/AKT signaling pathway, and then enhances HCC invasion through epithelial–mesenchymal transition and sorafenib resistance.^28^ This study is consistent with our findings on Gal-1 promoting epithelial–mesenchymal transition. In addition, our experiments also confirmed that Gal-1 could promote the proliferation of hepatoma cells.

Hypoxia, on the other hand, is an important feature of the tumor microenvironment in solid tumors, and it has been considered a major regulator of Gal-1 in tumors for a long time.^29^ HIFs are the major factors in cell response to hypoxia; it has been reported in earlier studies that HIF-1α can bind to the gene promoter region of Gal-1 in colorectal cancer cells and acute myeloid leukocytes, but the specific binding sites and regulatory mechanisms are different. In colon cancer cells, HIF-1α directly binds to Gal-1 on the gene transcription start site from −441 bp to −423 bp,^26^ while in acute myeloid leukocytes, HIF-1α and C/EBP-α co-regulate the expression of Gal-1 by binding to its gene transcription start site from −42 to −48 bp.^27^ These results suggest an important correlation between HIFs and Gal-1, but its specific regulatory mechanism may be diverse in different tumor cells. In our study, from the TCGA database information, we found that HIF-1α was also correlated with Gal-1 in HCC, but CUT&RUN experiments showed that HIF-1α did not directly bind to the gene promoter region of Gal-1 in hepatoma cells, suggesting other regulatory mechanisms may exist.

Recent studies have shown that a hypoxic microenvironment often causes the dysregulation of m^6^A methylation, which is closely related to the progression of HCC.^30^ Li et al have demonstrated that HIF-1α up-regulates m^6^A reader YTHDF1 under hypoxic conditions, then YTHDF1 promotes the transcription of ATG2A and ATG14, and in turn enhances the autophagy of hepatoma cells.^31^ A hypoxia-responsive lncRNA, AC115619, has also been found to encode a micropeptide called AC115619-22AA, which binds to WTAP and blocks the assembly of the m^6^A methyltransferase complex, and this process results in inhibiting HCC progression.^32^ These studies suggest that hypoxia affects HCC progression through a variety of molecules and different regulatory mechanisms that are related to m^6^A. However, is the expression of Gal-1 also affected by m^6^A modification?

In previous studies, Gal-1 has never been reported to be regulated by any m^6^A-modifying enzymes. For the first time, we speculate that the changes of Gal-1 under hypoxic conditions may be related to m^6^A modification. After full-length transcriptome sequencing of MHCC-97H cells under hypoxia, we noted significant changes in the RNA methylation pathway and mRNA binding pathway, as well as changes in the expression of multiple m^6^A modification-related enzymes. We then found a close association between ALKBH5/YTHDF2 with Gal-1 in HCC, and they were both associated with poor prognoses in HCC patients. However, the difference is that the high expression of ALKBH5 usually reduces the methylation level of mRNA, thus reducing the binding probability of the reader, while the decrease of YTHDF2 directly reduces its binding probability with mRNA. Both cases will reduce the efficiency of YTHDF2 transporting mRNA to the RNA degrader and improve the stability of mRNA. In this way, the expression of cancer-related genes was promoted.^33^ After testing the half-life of Gal-1 mRNA after knocking down ALKBH5 and overexpressing YTHDF2, the results confirmed that Gal-1 mRNA stability was reduced, which implies that the overproduction of Gal-1 in HCC is closely due to the demethylation of m^6^A modification. RNA immunoprecipitation-quantitative PCR verified the binding of ALKBH5 and YTHDF2 to Gal-1, and RMvar and SRAMP online tools were used to predict the binding sites, and dual luciferase assay identified the m^6^A modification site in Gal-1 5′-UTR.

Combined with the above conclusions, we found that hypoxia regulated ALKBH5 and YTHDF2, which in turn caused the change of Gal-1 expression. But how does hypoxia affect the m^6^A modification of HCC cells in the specific mechanism? HIFs, as the initiating sensor and effector of the hypoxia process, may provide the answer. In the next step, based on the specific sites predicted by JASPAR, dual luciferase assay was conducted to confirm HIF-1α′s binding to the promoter region of ALKBH5 and HIF-2α′s binding to the promoter region of YTHDF2. However, the effects they produced were opposite. HIF-1α positively regulated ALKBH5, while HIF-2α functioned as a transcription factor and inhibited the expression of YTHDF2. However, in sight of the disease, both the increase of ALKBH5 and the decrease of YTHDF2 promote the development of liver cancer.^20^^,^^21^ This trend reflects the complexity and synergy when hypoxia regulates hepatoma cells.

Notably, transcriptomic analysis of hypoxic HCC cells identified altered expression of multiple m^6^A-modifying enzymes. While our study focuses on ALKBH5 and YTHDF2, other enzymes may contribute to HCC pathogenesis through distinct mechanisms. Existing literature indicates that METTL3 is significantly up-regulated in HCC tissues and correlates with poor prognosis.^34^ Furthermore, METTL3 has been demonstrated to be regulated by hypoxia in colorectal cancer,^35^ non-small cell lung cancer,^36^ and renal cell carcinoma.^37^ Thus, it is plausible that hypoxia-driven dysregulation of METTL3 also promotes malignant transformation in HCC cells. The hypoxic regulation of FTO involves more complex pathways. FTO can be modulated by sirtuin 1 (SIRT1) to exert oncogenic effects via the G protein subunit alpha O1 (GNAO1) axis.^38^ Alternatively, it may inhibit degradation of glycoprotein non-metastatic melanoma protein B (GPNMB), thereby attenuating CD8^+^ T cell-mediated cytotoxicity against HCC cells.^39^ These findings collectively establish FTO as a critical role in HCC progression. It is noteworthy that hypoxic regulation of both SIRT1 and GPNMB has been partially documented.^40^^,^^41^ Probing FTO's potential role within these established pathways may yield deeper insights into the hypoxic tumor microenvironment of HCC.

A study published earlier found that the intervention of Gal-1 affected multiple pathways related to liver cancer tissue location using AAV9 to overexpress or silence Gal-1. These pathways include regulation of translational mechanisms within tumors, interactions between CD45 cells, tumor margin stroma formation, and metastatic T cell populations.^42^ In our study, we used shGal-1 MHCC-97H cells to establish a subcutaneous tumor model in nude mice, and found that the intervention of Gal-1 not only affected the proliferation markers like PCNA and KI67, but also epithelial–mesenchymal transition markers N-cadherin, E-cadherin, and Vimentin. LNP is a non-viral lipid-based delivery system, with the advantages of safety, tolerability, ability to re-dose, large genetic cargo, ease of design, and straightforward manufacturing processes. It may well become dominant.^43^ A recent study successfully ameliorated and reversed the progress of pulmonary fibrosis by encapsulating runt-related transcription factor-1 siRNA (siRUNX1) into lung-targeting LNP.^44^ In our study, we designed LNPs encapsulating Gal-siRNA and labeled them with Cy5. By electron microscopy and particle size analysis, we confirmed that LNP-siRNA was successfully encapsulated. IF and flow cytometry also showed that LNP-siRNA was successfully transfected into MHCC-97H, and *in vivo* imaging after tail vein injection of LNP-siRNA showed that LNP-siRNA could be successfully enriched in the mouse liver after tail vein injection of LNP-siRNA. Finally, immunofluorescence analysis confirmed that LNP-siRNA effectively targeted Gal-1 *in vivo*, and it decreased the expression of N-cadherin and Vimentin and increased the expression of E-cadherin, which further confirmed that Gal-1 could be a viable therapeutic target for HCC.

**Figure 9:**
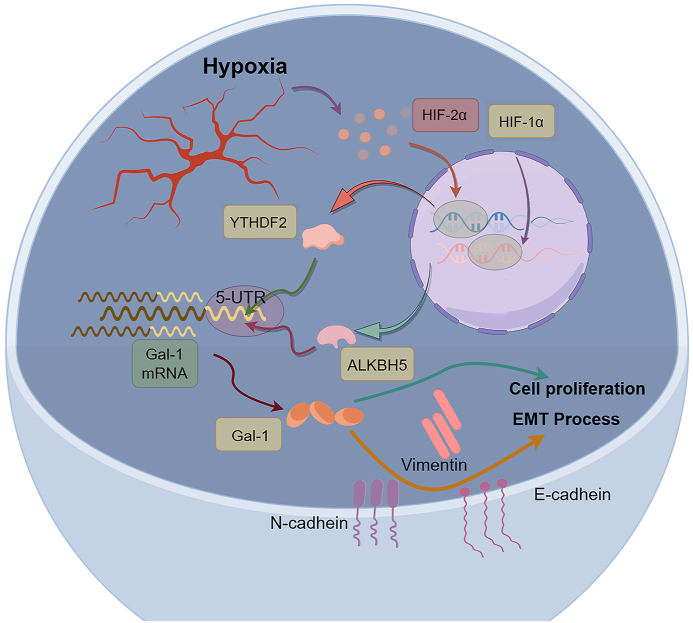
Schematic diagram of this study. HIF-1α regulates the expression of ALKBH5, and HIF-2α regulates the expression of YTHDF2; these mechanisms drive hypoxia-induced malignancy of hepatocellular carcinoma by promoting Gal-1 translation. In brief, the up-regulation of Gal-1 in HCC is positively related to ALKBH5 and YTHDF2.

In conclusion, our study highlighted the importance of Gal-1 as a therapeutic target for HCC, and we also explored the upstream regulatory mechanisms of Gal-1 in the hypoxia microenvironment of HCC. HIFs up-regulated the expression of Gal-1 through ALKBH5 and YTHDF2 in an m^6^A-dependent manner, driving the proliferation and epithelial–mesenchymal transition of hepatoma cells, and promoting the progression of HCC (Fig. 9).

## CRediT authorship contribution statement

**Xianzhi Gao:** Writing – review & editing, Writing – original draft, Visualization. **Bian Shu:** Formal analysis. **Diguang Wen:** Methodology. **Jiao Lu:** Supervision, Software. **Hua Song:** Validation, Conceptualization. **Ziyi Sheng:** Validation. **Yu You:** Resources. **Zuojin Liu:** Supervision, Resources, Project administration.

## Data availability

The original data can be obtained from the corresponding author with a reasonable request.

## Ethics declaration

All animal experiments were approved by the Animal Experimentation Ethics Committee of the Second Affiliated Hospital of Chongqing Medical University, Chongqing, China (approval number: IACUC-SAHCQMU-2025-0101). All of the experiments were performed in accordance with the Basel Declaration. Written informed consent was obtained from all patients, and the study was conducted by the local ethics committee following the rules of the Helsinki Declaration (No. 2020-CQMU-157).

## Funding

This work was funded by the 10.13039/501100001809National Natural Science Foundation of China (No. 82370672, 8207034238), Kuanren Talents Program of the second affiliated hospital of Chongqing Medical University (No. kryc-yq-2208), Chongqing Natural Science Foundation (No. CSTB2023NSCQ-MSX0150) and Chongqing Municipal Science and Health Joint Medical Research Project (No. 2024QNXM003).

## Conflict of interests

The authors declare that they have no potential financial conflicts of interest related to this manuscript. None of the material in this paper has been published or is under consideration for publication elsewhere.

## Figures and Tables

**figs1:**
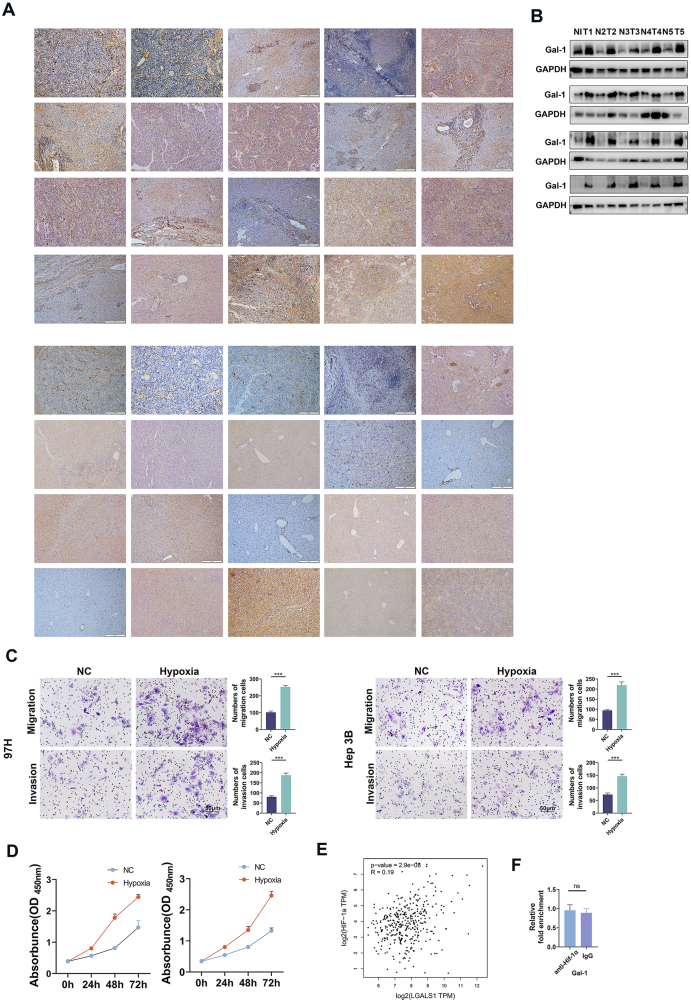


**figs2:**
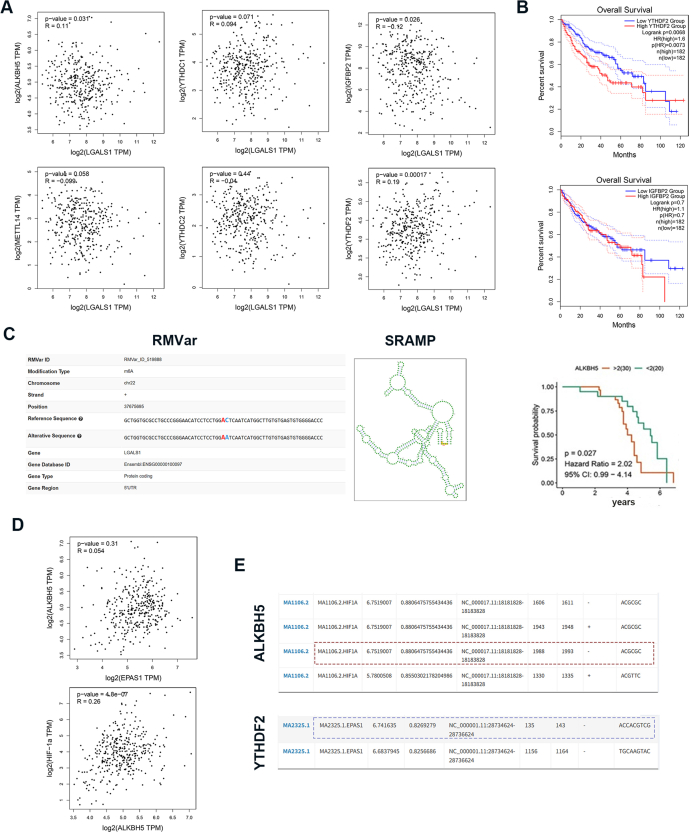


## References

[1] Zhang X., He C., He X. (2023). HIF-1 inhibitor-based one-stone-two-birds strategy for enhanced cancer chemodynamic-immunotherapy. J Contr Release.

[2] Xu Q., Liu X., Liu Z. (2017). MicroRNA-1296 inhibits metastasis and epithelial-mesenchymal transition of hepatocellular carcinoma by targeting SRPK1-mediated PI3K/AKT pathway. Mol Cancer.

[3] Sung H., Ferlay J., Siegel R.L. (2021). Global cancer statistics 2020: GLOBOCAN estimates of incidence and mortality worldwide for 36 cancers in 185 countries. CA Cancer J Clin.

[4] Jing X., Yang F., Shao C. (2019). Role of hypoxia in cancer therapy by regulating the tumor microenvironment. Mol Cancer.

[5] Laitakari A., Huttunen R., Kuvaja P. (2020). Systemic long-term inactivation of hypoxia-inducible factor prolyl 4-hydroxylase 2 ameliorates aging-induced changes in mice without affecting their life span. FASEB J.

[6] Pietras A., Katz A.M., Ekström E.J. (2014). Osteopontin-CD44 signaling in the glioma perivascular niche enhances cancer stem cell phenotypes and promotes aggressive tumor growth. Cell Stem Cell.

[7] Cramer T., Vaupel P. (2022). Severe hypoxia is a typical characteristic of human hepatocellular carcinoma: scientific fact or fallacy?. J Hepatol.

[8] Mariño K.V., Cagnoni A.J., Croci D.O., Rabinovich G.A. (2023). Targeting galectin-driven regulatory circuits in cancer and fibrosis. Nat Rev Drug Discov.

[9] Girotti M.R., Salatino M., Dalotto-Moreno T., Rabinovich G.A. (2020). Sweetening the hallmarks of cancer: galectins as multifunctional mediators of tumor progression. J Exp Med.

[10] Orozco C.A., Martínez-Bosch N., Guerrero P.E. (2018). Targeting galectin-1 inhibits pancreatic cancer progression by modulating tumor-stroma crosstalk. Proc Natl Acad Sci U S A.

[11] Ouyang J., Juszczynski P., Rodig S.J. (2011). Viral induction and targeted inhibition of galectin-1 in EBV^+^ posttransplant lymphoproliferative disorders. Blood.

[12] Liu F.T., Rabinovich G.A. (2005). Galectins as modulators of tumour progression. Nat Rev Cancer.

[13] Croci D.O., Cerliani J.P., Dalotto-Moreno T. (2014). Glycosylation-dependent lectin-receptor interactions preserve angiogenesis in anti-VEGF refractory tumors. Cell.

[14] Lin T.W., Chang H.T., Chen C.H. (2015). Galectin-3 binding protein and galectin-1 interaction in breast cancer cell aggregation and metastasis. J Am Chem Soc.

[15] Chung L.Y., Tang S.J., Sun G.H. (2012). Galectin-1 promotes lung cancer progression and chemoresistance by upregulating p38 MAPK, ERK, and cyclooxygenase-2. Clin Cancer Res.

[16] Laderach D.J., Gentilini L.D., Giribaldi L. (2013). A unique galectin signature in human prostate cancer progression suggests galectin-1 as a key target for treatment of advanced disease. Cancer Res.

[17] Bannoud N., Stupirski J.C., Cagnoni A.J. (2023). Circulating galectin-1 delineates response to bevacizumab in melanoma patients and reprograms endothelial cell biology. Proc Natl Acad Sci U S A.

[18] Stanley P. (2014). Galectin-1 pulls the strings on VEGFR2. Cell.

[19] Pan X.Y., Huang C., Li J. (2021). The emerging roles of m^6^A modification in liver carcinogenesis. Int J Biol Sci.

[20] You Y., Wen D., Zeng L. (2022). ALKBH5/MAP3K8 axis regulates PD-L1^+^ macrophage infiltration and promotes hepatocellular carcinoma progression. Int J Biol Sci.

[21] Hou J., Zhang H., Liu J. (2019). YTHDF2 reduction fuels inflammation and vascular abnormalization in hepatocellular carcinoma. Mol Cancer.

[22] Yamamoto T., Kanda A., Kase S., Ishida S. (2021). Hypoxia induces galectin-1 expression via autoinduction of placental growth factor in retinal pigment epithelium cells. Invest Ophthalmol Vis Sci.

[23] Wen D., Xiao H., Gao Y., Zeng H., Deng J. (2024). N6-methyladenosine-modified SENP1, identified by IGF_2_BP_3_, is a novel molecular marker in acute myeloid leukemia and aggravates progression by activating AKT signal via de-SUMOylating HDAC2. Mol Cancer.

[24] Brown Z.J., Tsilimigras D.I., Ruff S.M. (2023). Management of hepatocellular carcinoma: a review. JAMA Surg.

[25] Imamura H., Matsuyama Y., Tanaka E. (2003). Risk factors contributing to early and late phase intrahepatic recurrence of hepatocellular carcinoma after hepatectomy. J Hepatol.

[26] Zhao X.Y., Chen T.T., Xia L. (2010). Hypoxia inducible factor-1 mediates expression of galectin-1: the potential role in migration/invasion of colorectal cancer cells. Carcinogenesis.

[27] Zhao X.Y., Zhao K.W., Jiang Y., Zhao M., Chen G.Q. (2011). Synergistic induction of galectin-1 by CCAAT/enhancer binding protein alpha and hypoxia-inducible factor 1alpha and its role in differentiation of acute myeloid leukemic cells. J Biol Chem.

[28] Zhang P.F., Li K.S., Shen Y.H. (2016). Galectin-1 induces hepatocellular carcinoma EMT and sorafenib resistance by activating FAK/PI3K/AKT signaling. Cell Death Dis.

[29] Mirchandani A.S., Sanchez-Garcia M.A., Walmsley S.R. (2025). How oxygenation shapes immune responses: emerging roles for physioxia and pathological hypoxia. Nat Rev Immunol.

[30] Zhang F., Liu H., Duan M. (2022). Crosstalk among m^6^A RNA methylation, hypoxia and metabolic reprogramming in TME: from immunosuppressive microenvironment to clinical application. J Hematol Oncol.

[31] Li Q., Ni Y., Zhang L. (2021). HIF-1α-induced expression of m6A reader YTHDF1 drives hypoxia-induced autophagy and malignancy of hepatocellular carcinoma by promoting ATG2A and ATG14 translation. Signal Transduct Target Ther.

[32] Zhang Q., Wei T., Yan L. (2023). Hypoxia-responsive lncRNA AC115619 encodes a micropeptide that suppresses m^6^A modifications and hepatocellular carcinoma progression. Cancer Res.

[33] Deng X., Qing Y., Horne D., Huang H., Chen J. (2023). The roles and implications of RNA m^6^A modification in cancer. Nat Rev Clin Oncol.

[34] Liu B., Cao J., Wu B. (2023). METTL3 and STAT3 form a positive feedback loop to promote cell metastasis in hepatocellular carcinoma. Cell Commun Signal.

[35] Yang Z., Quan Y., Chen Y. (2021). Knockdown of RNA N^6^-methyladenosine methyltransferase METTL3 represses Warburg effect in colorectal cancer via regulating HIF-1α. Signal Transduct Target Ther.

[36] Yang Y., Cheng C., He B. (2023). Cigarette smoking, by accelerating the cell cycle, promotes the progression of non-small cell lung cancer through an HIF-1α-METTL3-m^6^A/CDK2AP2 axis. J Hazard Mater.

[37] Chen Y., He Y., Li Z. (2024). METTL3 facilitates renal cell carcinoma progression by PLOD2 m^6^A-methylation under prolonged hypoxia. Cell Death Dis.

[38] Liu X., Liu J., Xiao W. (2020). SIRT1 regulates N^6^-methyladenosine RNA modification in hepatocarcinogenesis by inducing RANBP2-dependent FTO SUMOylation. Hepatology.

[39] Heinrich B., Cubero F.J. (2024). FTO/m6A/GPNMB axis: a novel promising target for hepatocellular carcinoma (HCC) treatment?. Gut.

[40] Shin D.H., Choi Y.J., Park J.W. (2014). SIRT1 and AMPK mediate hypoxia-induced resistance of non-small cell lung cancers to cisplatin and doxorubicin. Cancer Res.

[41] Zhang J., Hu S., Jin X. (2025). Hypoxia-associated GPNMB^+^ macrophages promote malignant progression of colorectal cancer and its related risk signature are powerful predictive tool for the treatment of colorectal cancer patients. Environ Toxicol.

[42] Setayesh T., Hu Y., Vaziri F. (2024). Targeting stroma and tumor, silencing galectin 1 treats orthotopic mouse hepatocellular carcinoma. Acta Pharm Sin B.

[43] Cullis P.R., Felgner P.L. (2024). The 60-year evolution of lipid nanoparticles for nucleic acid delivery. Nat Rev Drug Discov.

[44] Cheng M., Yu X., Qi S. (2024). Development of organ-targeting lipid nanoparticles with low immunogenicity and their application in the treatment of pulmonary fibrosis. Angew Chem Int Ed.

